# Nuclear Quantum Effects Reshape Structural Signatures of Supercooled Water near the Liquid–Liquid Critical Region

**DOI:** 10.1021/acs.jpcb.6c03267

**Published:** 2026-08-15

**Authors:** M. Beerbaum, J. Heske, J. Gujt, Thomas D. Kühne

**Affiliations:** † Center for Advanced Systems Understanding (CASUS), Conrad-Schiedt-Straße 20, Görlitz 02826, Germany; ‡ Helmholtz Zentrum Dresden-Rossendorf, Bautzner Landstraße 400, Dresden 01328, Germany; § Chair of Theoretical Chemistry and Center for Sustainable Systems Design, Paderborn University, Warburger Str. 100, Paderborn D-33098, Germany; ∥ Institute of Artificial Intelligence, Technische Universität Dresden, Helmholtzstraße 10, Dresden 01069, Germany

## Abstract

The liquid–liquid transition scenario for supercooled water relies heavily on structural markers that distinguish low-density-liquid-like from high-density-liquid-like environments. Because these markers are often evaluated with classical nuclei, it remains unclear how zero-point motion changes the interpretation of the putative liquid–liquid critical region. Here, we compare classical molecular dynamics (MD) and path-integral molecular dynamics (PIMD) simulations of a flexible q-TIP4P/F-like water model over temperatures and pressures spanning the thermodynamic region where this model is expected to exhibit a liquid–liquid critical point. Classical trajectories display a sharp density increase at 180 K between 180 and 220 MPa, whereas path-integral simulations give a smoother pressure response. Nuclear quantum effects (NQE) broaden oxygen–oxygen, oxygen–hydrogen, and hydrogen–hydrogen correlations and reduce the first-shell tetrahedral order, yet they slightly increase the nearest-neighbor Steinhardt *Q*_6_ parameter. Thus, quantum nuclei do not simply blur all structural signatures uniformly; they renormalize different order parameters in different directions. These results identify nuclear quantum motion as a necessary ingredient when assigning LDL-like and HDL-like structural motifs and caution against sharp two-state assignments based on static classical order parameters alone.

## Graphical abstract

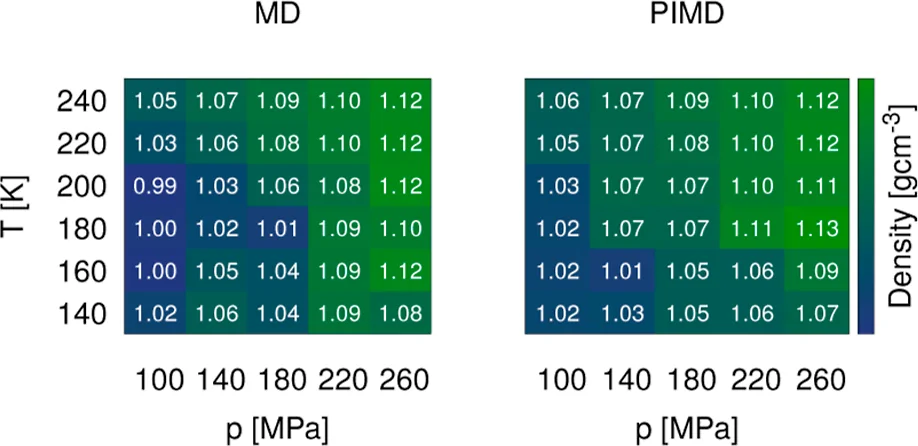

## Introduction

Water is one of the most extensively studied molecular liquids, yet the microscopic origin of many of its anomalies remains incompletely understood.^1^ In the supercooled regime, thermodynamic response functions such as the isobaric heat capacity and the isothermal compressibility increase rapidly below the homogeneous ice-nucleation temperature *T*_H_.^2,3^ A widely discussed interpretation attributes this behavior to a metastable first-order liquid–liquid phase transition (LLPT) between low-density liquid (LDL) and high-density liquid (HDL) water, ending in a liquid–liquid critical point (LLCP).^4^

Direct experimental access to this region is difficult because rapid crystallization occurs below *T*_H_, which is why it is often referred to as water’s no man’s land. Experimental strategies to circumvent crystallization include aqueous solutions,^5–9^ confined water,^9–12^ and nanodroplet experiments.^13,14^ In parallel, molecular simulations have provided detailed microscopic information on the metastable liquid. Poole et al. first proposed a LLPT for the ST2 water model,^15^ and Palmer et al. later estimated the corresponding LLCP at 230 K and 240 MPa.^16^ For TIP4*P*/2005 water, Abascal and Vega reported a LLCP at 193 K and 135 MPa,^17^ while subsequent studies located it near 182 K and 158–162 MPa.^18,19^ The existence and interpretation of the LLPT remain debated, with alternative explanations emphasizing finite-size effects and local ice-like ordering.^20–25^

Most earlier simulations of the LLPT used classical nuclei. This approximation is not obviously benign for water, where proton delocalization and zero-point motion can influence hydrogen-bond structure and dynamics. Previous path-integral molecular dynamics (PIMD)/RPMD and related simulations of ambient liquid water have shown that nuclear quantum effects (NQE) soften the liquid structure and modify translational, vibrational, and hydrogen-bond dynamics.^26–28^ Recently, Eltareb, Lopez, and Giovambattista used path-integral simulations and a potential-energy-landscape equation of state for q-TIP4P/F water to show that NQE shift the liquid–liquid critical point to lower temperature and pressure and modify the curvature of the landscape basins near the critical region.^29^ That thermodynamic study did not analyze the structural order parameters commonly used to assign LDL-like and HDL-like local environments. The present work is therefore complementary: we focus directly on how NQE reshape radial distribution functions and orientational order descriptors in the same region of the phase diagram.

The use of an empirical flexible water model also defines the scope of our conclusions. Modern machine-learned interatomic potentials have enabled first-principles-based studies of supercooled water and have provided evidence for a liquid–liquid transition in an ab initio deep-neural-network model.^30^ Such models are increasingly attractive for revisiting selected state points with improved energetics. Our goal here is not to establish a definitive equation of state for water but to isolate a descriptor-level question within a well-defined and computationally tractable q-TIP4P/F-like model: whether quantum nuclei change the structural markers that are often interpreted as LDL-like or HDL-like. In this work, we therefore ask how quantum nuclei reshape the structural markers used in the LLPT discussion, rather than attempting a definitive localization of the critical point. We compare classical MD with PIMD simulations using the flexible q-TIP4P/F-like model of Spura et al.^26^ We analyze densities, radial distribution functions (RDFs), the local tetrahedral order parameter *q*_4_,^31,32^ and the Steinhardt order parameter *Q*_6_ ^33,34^ to determine how NQE affect the structural distinction between LDL-like and HDL-like states.

## Methods

### Path-Integral Molecular Dynamics

Equilibrium PIMD exploits the classical isomorphism of discretized quantum statistical mechanics^35^ and maps each quantum nucleus onto a classical ring polymer of *P* beads connected by harmonic springs.^36^ In the limit *P* → ∞, this representation samples the exact quantum–mechanical equilibrium distribution. Up to normalization constants, the discretized canonical partition function can be written as

1 $$Z_{P}\propto \int dpdr\exp\left[-\beta_{P}H_{P}\left(p,r\right)\right]$$

where β_P_ = β/*P*. The ring-polymer Hamiltonian is

2 $$H_{P}=\sum_{k=1}^{P}\left[\sum_{i=1}^{N}\left(\frac{p_{i,k}^{2}}{2m_{i}}+\frac{1}{2}m_{i}\omega_{P}^{2}|r_{i,k}-r_{i,k+1}{|}^{2}\right)+V\left(r_{1,k},...,r_{N,k}\right)\right]$$

with ω_P_ = *P*/(βℏ) and cyclic boundary conditions in the bead index. Because the intermolecular interactions have to be evaluated for the ring-polymer representation, PIMD is more expensive than classical MD. We reduce this cost using the ring-polymer contraction approach,^37^ in which the expensive long-range electrostatic contribution is evaluated on the centroid, while the intramolecular terms retain the full bead resolution. Related auxiliary-potential contraction schemes have enabled ab initio PIMD of liquid water at nearly classical simulation cost.^38^

### Structural Order Parameters

Local tetrahedrality is quantified using the bond-orientational order parameter introduced by Chau and Hardwick^31^ and later modified by Errington and Debenedetti^32^

3 $$q_{i}=1-\frac{3}{8}\sum_{1\leq j<k\leq 4}{\left(\cos\theta_{jik}+\frac{1}{3}\right)}^{2}$$

where θ_jik_ is the angle formed by the vectors from molecule *i* to two of its four nearest neighbors. A perfectly tetrahedral environment gives *q*_i_ = 1, while uncorrelated configurations average to zero. The system-averaged tetrahedral order is

4 $$q_{4}=N^{-1}\sum_{i=1}^{N}q_{i}$$

To characterize structural order beyond the first hydration shell, we also compute a nearest-neighbor version of the Steinhardt order parameter^33,34^

5 $$Q_{l,i}=\sqrt{\frac{4\pi }{2l+1}\sum_{m=-l}^{l}{\left|{\mathrm{Y̅}}_{lm}\left(i\right)\right|}^{2}}$$

where ${\mathrm{Y̅}}_{lm}\left(i\right)$ is the average of the spherical harmonic *Y*_lm_ over the bond vectors connecting molecule *i* with its 12 nearest water molecules. This differs from the original Steinhardt definition, which averages over all neighbors inside a cutoff radius.^33^ We use a fixed nearest-neighbor definition to keep the number of bonds constant across density-changing state points; consequently, the absolute magnitude of *Q*_6_ should be interpreted together with this neighbor convention. We focus on *l* = 6 and use

6 $$Q_{6}=\frac{1}{N}\sum_{i=1}^{N}Q_{6,i}$$

For an ideal-gas reference state, denoted by the superscript “ig”, the *k* bond directions are uncorrelated and $Q_{6}^{ig}=1/\sqrt{k}$ , which gives *Q*_6_^ig^ ≈ 0.289 for *k* = 12.^39^ Values for crystalline reference structures are larger, for example, 0.574 for fcc, 0.484 for hcp, and 0.510 for bcc environments.^40^

### Simulation Details

All simulations were performed in the isothermal–isobaric ensemble using periodic supercells containing 125 water molecules. Classical MD and PIMD trajectories were generated with an in-house MD/PIMD implementation for the flexible q-TIP4P/F-like force field. The code propagates the explicit ring-polymer degrees of freedom, evaluates the q-TIP4P/F-like intramolecular and intermolecular forces, and applies the centroid contraction of the electrostatic contribution described above. Interactions were described with the q-TIP4P/F-like flexible water model of Spura et al.,^26^ which was parametrized by force matching to DFT-based TPSS-D3 reference calculations.^41^ This model is based on the q-TIP4P/F model of Habershon et al.,^42^ which in turn was derived from the rigid TIP4*P*/2005 model of Abascal and Vega.^43^ Unlike rigid TIP4*P*/2005, the q-TIP4P/F-like model includes intramolecular harmonic bending and anharmonic Morse stretching terms and is therefore suitable for simulations with quantum nuclei.

Each trajectory was pre-equilibrated for 0.5 ns in the canonical ensemble, followed by 3 ns of equilibration in the isothermal–isobaric ensemble. Production trajectories of 10 ns were then used for analysis. Temperature was controlled with an Andersen thermostat,^44^ and pressure was controlled with a Berendsen barostat.^45^ The Berendsen barostat was used to stabilize the extensive grid of deeply supercooled state points and to compare classical and quantum-nuclear simulations under identical coupling conditions. Because it does not generate the exact isothermal–isobaric ensemble and suppresses volume fluctuations, we do not use the present simulations to extract compressibilities, critical exponents, or a precise equation of state. The density data are used only as context for selecting representative LDL-like and HDL-like states, while the main conclusions are based on structural descriptors.

Multiple time-step integration^46^ used an inner time step of 0.25 fs for the stiff intramolecular and ring-polymer spring forces and an outer time step of 0.5 fs for the intermolecular forces. The temperature was varied from 140 to 240 K in steps of 20 K, and the pressure was varied from 100 to 260 MPa in steps of 40 MPa. PIMD simulations used *P* = 64 beads; otherwise, identical classical reference simulations were carried out with *P* = 1.

## Results and Discussion

### Density Response in the Liquid–Liquid Transition Region

Figure 1 shows the average densities obtained from classical MD and PIMD as a function of temperature and pressure. The classical simulations display a pronounced density increase at 180 K between 180 and 220 MPa, from approximately 1.0 to 1.1 g cm^–3^. The density time traces at 180 K fluctuate around stable mean values of about 1.01 and 1.10 g cm^–3^ on the low- and high-pressure sides, respectively. This behavior is consistent with a transition between LDL-like and HDL-like liquid states in the classical simulations.

**Figure 1:**
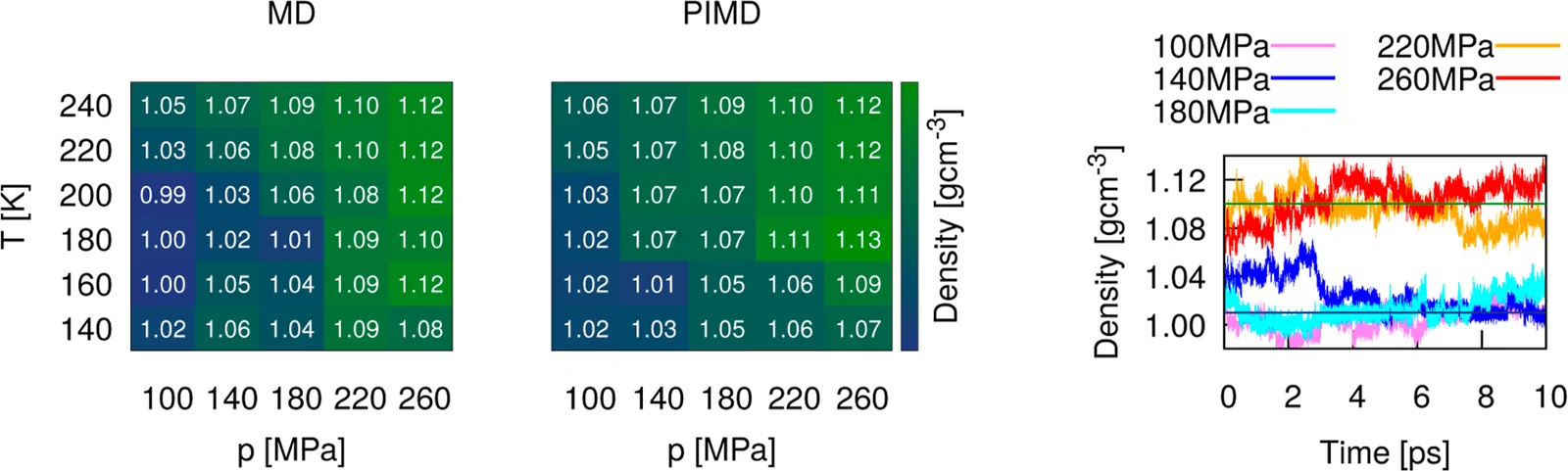
Average densities of the MD and PIMD simulations of water around the putative LLCP region (left) and density time traces from the classical MD simulations at 180 K for the five pressures considered (right).

The PIMD simulations do not show an equally sharp density discontinuity over the same grid of the thermodynamic states. Instead, the density increases more smoothly with the pressure in the low-temperature regime. This difference may indicate that the NQE shift, weaken, or remove the first-order transition in this model; alternatively, a remaining transition may not be resolvable from the present finite set of 10 ns trajectories because density is a slowly converging quantity in deeply supercooled water. The nonmonotonic temperature dependence visible in the classical density surface should be interpreted in the same spirit: the grid crosses metastable LDL-like and HDL-like regions rather than a single smoothly equilibrated liquid branch, so ordinary thermal expansion intuition need not apply point by point.

The finite system size and trajectory length are sufficient for the local structural comparison pursued here because RDFs and local bond-order distributions average over all molecules and many saved configurations. They are not sufficient for a finite-size scaling analysis or for a definitive statement about the existence, location, or disappearance of the LLPT. We therefore use the density data as a thermodynamic context for selecting representative LDL-like and HDL-like state points while focusing the main analysis on a more robust question: how quantum nuclei change the structural observables commonly used to identify these environments.

### Radial Distribution functions

To analyze how NQE change the microscopic structure, we compare RDFs for two representative state points at 180 K. The 100 MPa simulation is used as an LDL-like state and the 220 MPa simulation is used as an HDL-like state, based on the density jump observed in the classical simulations. Figure 2 shows the oxygen–oxygen, oxygen–hydrogen, and hydrogen–hydrogen RDFs for classical MD and PIMD.

**Figure 2:**
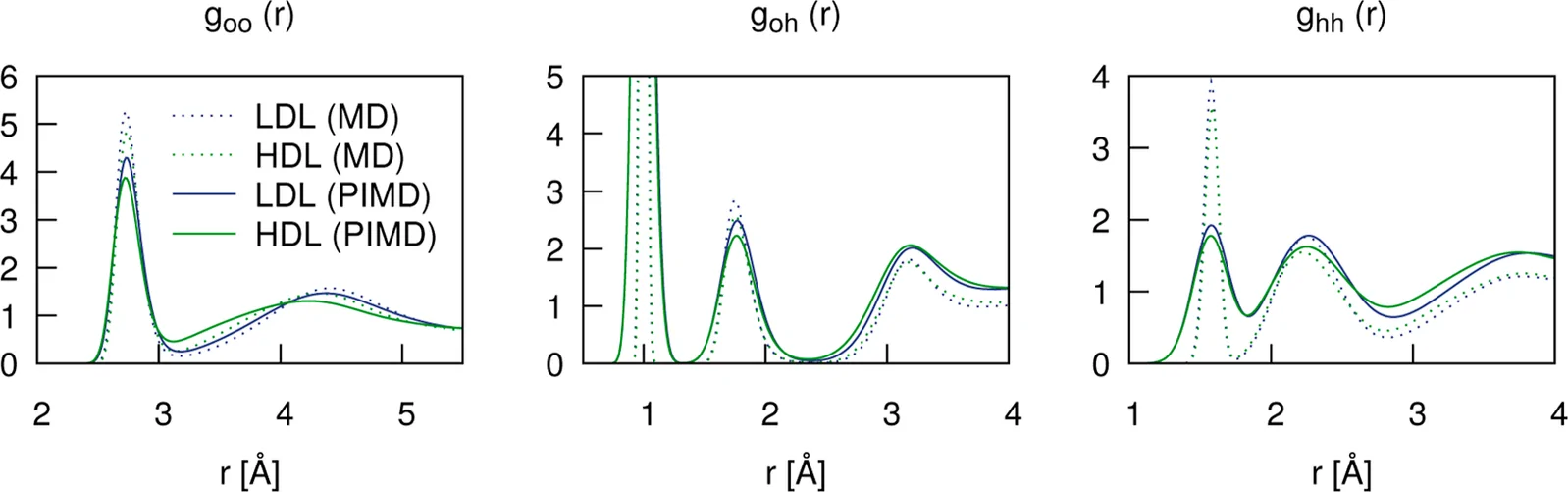
Effect of NQE on the radial distribution functions *g*_OO_(*r*), *g*_OH_(*r*), and *g*_HH_(*r*) of LDL-like and HDL-like water at 180 K. The RDFs are compared between PIMD and classical MD.

Including NQE lowers the peak heights, broadens the peaks, and makes the RDFs generally less structured. This broadening is expected from the zero-point motion of the nuclei and is most pronounced in pair correlations involving hydrogen. The oxygen–oxygen RDF is affected more weakly, consistent with the larger mass of oxygen. The HDL-like RDFs are shifted to slightly shorter distances relative to the LDL-like RDFs, reflecting the higher density of the HDL-like state. Overall, the RDFs show that NQE do not merely perturb the hydrogen coordinates locally; they soften the pair structure of both LDL-like and HDL-like liquid water in the LLPT region.

### Bond-Order Parameters

The trends observed in the RDFs are reflected in the bond-order parameters shown in Figure 3. The average tetrahedral order parameter *q*_4_ lies between 0.75 and 0.83 across the investigated state points. These values exceed reported values of 0.6686 at 298 K and 0.7297 at 260 K for ambient-pressure liquid water,^47^ consistent with the enhanced tetrahedral ordering expected in deeply supercooled water.

**Figure 3:**
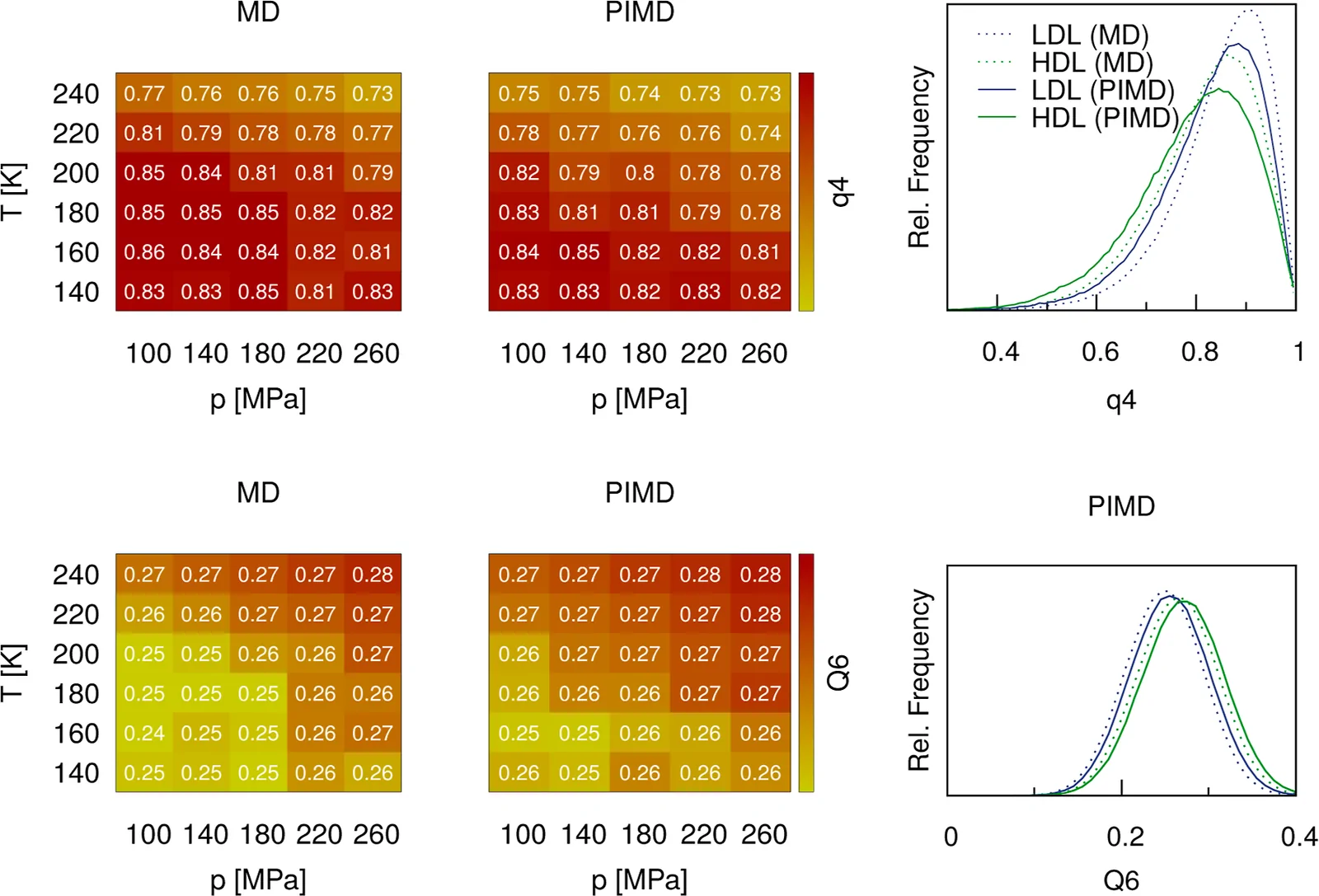
Effect of NQE on the tetrahedral order parameter *q*_4_ (upper panels) and the Steinhardt parameter *Q*_6_ (lower panels). The left panels show averages over the investigated thermodynamic state points, while the right panels show distributions for representative LDL-like and HDL-like states.

Temperature has the strongest influence on *q*_4_: lowering the temperature increases the tetrahedral order until the trend reaches a maximum or plateau around 180 K. At the representative 180 K state points, HDL-like water is less tetrahedral than LDL-like water. PIMD systematically lowers *q*_4_ relative to the corresponding classical simulations, demonstrating that NQE reduce the first-shell tetrahedral order. This reduction is also visible in the distributions of local *q*_*i*_ values, which shift toward lower tetrahedralities when quantum nuclei are included.

The Steinhardt *Q*_6_ parameter provides complementary information on ordering extending toward the second hydration shell. In contrast to *q*_4_, *Q*_6_ increases with increasing temperature and pressure in the present simulations. The values remain close to the uncorrelated reference value *Q*_6_^ig^ ≈ 0.289 for 12 neighbors, especially at higher temperatures and pressures. Thus, while the first shell remains strongly tetrahedral, the second-shell ordering is far from crystalline. Because *Q*_6_ depends on the neighbor definition used in the analysis,^48^ its absolute value should not be overinterpreted; the robust information is the relative trend across state points and between classical and quantum nuclei.

The *Q*_6_ distributions show a small shift to higher values in PIMD simulations. This NQE-induced shift is weaker than the corresponding decrease in *q*_4_, indicating that quantum nuclei most strongly disrupt first-shell tetrahedrality while producing only a modest reorganization of the more extended local environment. Together, the *q*_4_ and *Q*_6_ results provide the central physical message of this work: NQE do not produce a single, uniform loss of order but renormalize commonly used structural descriptors in a descriptor- and length-scale-dependent manner.

These results also bear on structural two-state interpretations of water, in which anomalous thermodynamics are linked to fluctuations between locally HDL-like and LDL-like environments.^49^ Our simulations do not rule out such thermodynamic descriptions, but they sharpen the requirement that structural evidence for two-state behavior be evaluated with quantum nuclei included. The distinction between LDL-like and HDL-like water is sensitive to the structural descriptor: *q*_4_ suggests reduced first-shell tetrahedrality upon including NQE, whereas *Q*_6_ changes in the opposite direction and remains close to the uncorrelated reference. A related caveat applies to inherent-structure analyses, where energy minimization removes thermal and quantum fluctuations and can sharpen bimodality in local-structure distributions.^50^ The NQE-induced broadening of pair correlations and reduction of tetrahedral order therefore caution against interpreting static structural order parameters as evidence for sharply separated local LDL- and HDL-like species, particularly when nuclear quantum motion is neglected.

## Conclusions

We have compared classical MD and PIMD simulations of flexible q-TIP4P/F-like water in the deeply supercooled region around the putative LLCP. The classical simulations show a pronounced density change at 180 K between 180 and 220 MPa, consistent with a transition between LDL-like and HDL-like liquid states. In contrast, the PIMD simulations show a smoother pressure dependence over the same state-point grid, in qualitative agreement with the recent potential-energy-landscape work showing that NQE shift the q-TIP4P/F liquid–liquid critical region.^29^

The structural analysis shows that NQE broaden the RDFs, reduce first-shell tetrahedrality, and slightly increase the nearest-neighbor Steinhardt *Q*_6_ parameter. These changes occur in both LDL-like and HDL-like states, demonstrating that NQE are not a small correction in the no man’s land regime. More importantly, the opposite trends in *q*_4_ and *Q*_6_ show that quantum nuclei alter the physical meaning of static order parameters used to assign local water environments. The present simulations do not determine the LLCP location precisely, but they establish that structural interpretations of the LLPT with water models of this type should include quantum nuclei. Longer trajectories, larger systems, and selected cross-checks with modern machine-learned potentials will be required to distinguish a shifted LLPT from a genuine smoothing or disappearance of the transition.
